# Problematic Smartphone Use during the COVID-19 Pandemic: Its Association with Pandemic-Related and Generalized Beliefs

**DOI:** 10.3390/ijerph18115724

**Published:** 2021-05-26

**Authors:** Meng Xuan Zhang, Juliet Honglei Chen, Kwok Kit Tong, Eilo Wing-yat Yu, Anise M. S. Wu

**Affiliations:** 1 Department of Psychology, Faculty of Social Sciences, University of Macau, Macao, China; yb77304@um.edu.mo (M.X.Z.); JulietChen@um.edu.mo (J.H.C.); kktong@um.edu.mo (K.K.T.); 2 Centre for Cognitive and Brain Sciences, Institute of Collaborative Innovation, University of Macau, Macao, China; 3 Department of Government and Public Administration, Faculty of Social Sciences, University of Macau, Macao, China; eiloyu@um.edu.mo

**Keywords:** COVID-19, pandemic, problematic smartphone use, pandemic-related beliefs, generalized social beliefs, social axioms

## Abstract

Smartphone technologies have played a crucial role in the fight against the COVID-19 pandemic; however, the increased use of smartphones during the pandemic period may expose the general public to a higher risk of problematic smartphone use (PSU). This study aimed to estimate the prevalence of PSU among Chinese community adults and adopted a social-cognitive theory and social axiom framework to evaluate the effects of beliefs on PSU. A Chinese adult sample (*N* = 616) was obtained through probability sampling via a telephone survey from Macao, China and included 591 smartphone users’ data (39.4% men) for formal analysis. The prevalence of PSU was 43.3% in the overall sample, with 41.9% in women, and 45.5% in men. Two types of beliefs derived from the social-cognitive theory, pandemic-related self-efficacy and government efficacy, both showed significant and negative correlations with PSU (*r* = −0.13 and −0.10, *p* < 0.05). As for the two beliefs from the social axiom framework, reward for application was negatively correlated with PSU (*r* = −0.10, *p* < 0.05), whereas social cynicism was positively associated with PSU (*r* = 0.25, *p* < 0.001). Among those four beliefs, social cynicism exerted the most substantial effect on PSU when controlling for demographics. Our findings enriched the understanding of PSU during the pandemic and provided empirical direction regarding cognition-based intervention strategies for reducing PSU.

## 1. Introduction

Coronavirus 2019 (COVID-19) is a highly infectious disease trigged by the severe acute respiratory syndrome coronavirus 2 (SARS-CoV-2). Since its first outbreak at the end of 2019, it quickly developed into a public health emergency of international concern by 31 January 2020 and was upgraded to a pandemic on 11 March 2020 [1]. Smartphone technologies have played an essential role in combatting the COVID-19 pandemic by offering a way to disseminate COVID-19 related information, record symptoms, and [2] trace potentially infected contacts [3], as well as to communicate, obtain and share information, and bring people together in times of enforced social isolation during the pandemic [4]. Meanwhile, it is not surprising that researchers observed a significant increase in smartphone use to access social networking, the Internet, and entertainment applications during the pandemic [5,6]. Despite the merits associated with smartphone technologies, researchers have warned that a surge in smartphone use may lead to an increase in problematic smartphone use (PSU) during the COVID-19 pandemic [7].

PSU is defined as excessive, addictive, and/or inappropriate smartphone use, with accompanying risks to physical and mental health, as well as social impairment in daily living [8,9]. Apart from the increased need to bridge social distancing and stay informed with smartphones, PSU may be exacerbated by the sizable prevalence of psychological distress in various communities during the pandemic [10,11]. Mental distress can be both an antecedent and a consequence of addictive behaviors in different populations [12,13,14,15]. Therefore, it should not be surprising that emerging findings have shown a positive correlation between COVID-19 anxiety and PSU severity [7]. However, no study has yet examined the effect of cognitions on PSU during the COVID-19 pandemic. Because cognitions, such as beliefs, have the potential to relieve one’s mental distress in general [16], they may also have the potential to lower one’s propensity for PSU. Therefore, the present study aimed to investigate the prevalence of PSU during the pandemic and its association with pandemic-related beliefs (i.e., perceived self-efficacy to adhere to precautionary measures and perceived government efficacy for combating the COVID-19 pandemic) as well as generalized social beliefs (i.e., social axioms) among community-dwelling adults in Macao, China. In the following, we first provided a literature review to build up the theoretical framework of pandemic-related beliefs and generalized beliefs with PSU, respectively, and then put forward four specific hypotheses regarding the hypothesized relations between a specific belief and PSU.

### 1.1. Pandemic-Related Beliefs and PSU

According to social-cognitive theory [17], people’s expectations of their ability to carry out an action (i.e., perceived self-efficacy) is a key determinant of their performing the action. Both generalized self-efficacy and behavior-specific self-efficacy have been found to be negatively correlated with health-compromising behaviors (e.g., smoking and poor dietary habits [18]) and adverse mental health conditions (e.g., collective trauma, anxiety, and depression [19,20,21]). Previous research has also shown the positive effects of behavior-specific self-efficacy on not only preventive behaviors like influenza vaccination [22,23] but also improved mental health in general [24]. In particular, the behavioral addiction field has identified behavior-specific self-efficacy as a protective factor for various types of addictive behaviors, such as problematic gambling [25,26] and problematic Internet use [27]. Although there is a handful of empirical support to the impact of specific self-efficacy on addictive behaviors, no research has yet explored the relationship between specific self-efficacy and PSU under the pandemic. Because situational factors are vital to one’s notion of self-efficacy concerning specific tasks [28], it is important to investigate self-efficacy in the pandemic context and locate specific types of pandemic-related efficacy related to PSU.

Because of its high contagiousness and potentially severe medical consequences, the COVID-19 pandemic is a multi-faceted stressful event that challenges one’s coping system. Adherence to personal precautionary measures, such as washing hands and wearing face masks, appears to be an adaptive coping mechanism with the pandemic in terms of reducing one’s susceptibility to contracting the disease [2] and has the potential to lower psychological distress during the pandemic [29]; conversely, excessive smartphone use (i.e., PSU) is one of the malfunctional coping approaches that would eventually disturb one’s daily functioning, despite its temporary distress relief at the beginning [30]. The self-efficacy that occurs due to following COVID-19 precautionary measures (hereinafter pandemic-related self-efficacy) appears to be crucial in determining individual differences regarding adherence to these measures (e.g., [31,32]). Specifically, those with higher levels of pandemic-related self-efficacy tend to be more likely to adopt adaptive coping and follow COVID-19 precautionary measures (e.g., face mask use), and thus less likely to resort to PSU as a malfunctional coping, and vice versa. Therefore, we hypothesized a negative correlation between pandemic-related self-efficacy and PSU (H1); in other words, people with low pandemic-related self-efficacy may have a higher risk for PSU in terms of reporting more PSU symptoms.

The perceived efficacy of the government’s intervention strategies against COVID-19 (hereinafter perceived pandemic-related government efficacy) is another potential protective factor against PSU during the pandemic. Like many other governments that have taken active pandemic-related measures, the Macao government has implemented a temporary city lockdown, provided a sufficient face mask supply, recommended work and study from home, and reduced public activities [33]. People may evaluate the effectiveness of these governmental actions differently and hence differ in their perceived government efficacy regarding COVID-19 control. Although no previous study has examined the relationship between perceived pandemic-related government efficacy and PSU, it is plausible that a higher level of perceived pandemic-related government efficacy would be associated with a lower risk of PSU. Because greater trust in the government to carry out effective measures to fight against this pandemic tends to increase one’s compliance with government recommended measures [34,35], this lowers the likelihood to practice malfunctional copings like PSU. Furthermore, a higher level of trust in governmental actions has been found to be associated with a lower level of mental health burden [36]; therefore, a higher level of perceived pandemic-related government efficacy may similarly buffer mental distress and associated health conditions (e.g., PSU) during the COVID-19 pandemic period. Therefore, we hypothesized that perceived pandemic-related government efficacy would negatively correlate with PSU (H2).

### 1.2. Generalized Social Beliefs and PSU

Social axioms refer to generalized social beliefs with respect to oneself and the environment [37]. According to Leung et al.’s findings [37,38], there are five social axioms that are independent, stable, and universally found cross-culturally, namely: social cynicism, reward for application, fate control, religiosity, and social complexity. Each social axiom can influence people’s perceptions and reactions towards daily life events and are accompanied with emotional experiences. Among the five social axioms, social cynicism and reward for application are especially relevant to our study. Although no research has yet studied how social axioms are related to PSU, an extant study has revealed that social cynicism and reward for application both had a statistically significant association with psychological wellbeing [39], indicating their potential impact on PSU during this pandemic.

Social cynicism refers to negative beliefs regarding the social world and human nature and specifically includes biases against certain social groups and mistrust in authorities [38]. It predicts poor mental health, such as suicidal behavior, loneliness, and dissatisfaction in life [40,41]. In the field of addictive behaviors, one study found a higher level of social cynicism was associated with a higher gambling propensity [42], and we may infer a similarly positive association between social cynicism and PSU. Based on the conceptualization of social cynicism, people with a higher level of social cynicism tend to attribute problems to external situations (i.e., environment) and/or others, and hence are more vulnerable to maladaptive coping and wishful thinking [43]. During the pandemic, these people may also be at a greater risk for PSU in terms of using smartphones excessively as a form of maladaptive coping with stressful contexts or mental distress [44]. Social cynicism was, therefore, hypothesized to be positively related to PSU (H3).

Reward for application is the belief that one’s efforts, such as careful planning and the investment of resources, will result in positive outcomes [37]. People with a higher level of reward for application tend to actively apply problem-solving coping strategies and make continuous efforts to change their future and achieve life goals, especially in the face of challenges and failure in their lives [43]. Accumulating empirical evidence has supported the protective role of reward for application in buffering the negative effects that adverse life events may have on psychological distress and suicidal ideation [45], and its facilitating effect of predicting flourishing among Chinese people [39]. Similarly, we expect that reward for application may play a protective role against PSU during the pandemic, and hence hypothesized reward for application would be negatively correlated with PSU in this study (H4).

### 1.3. The Present Study

This study aimed to first estimate the prevalence of PSU during the pandemic, and then, based on a social-cognitive theory and social axioms framework, to investigate associations between pandemic-related beliefs, as well as generalized social beliefs, and PSU. Four specific hypotheses were put forward: a negative correlation was postulated for PSU with pandemic-related self-efficacy (H1), perceived pandemic-related government-efficacy (H2), and reward for application (H4); while social cynicism was hypothesized to be positively associated with PSU (H3). To be more precise, we expected those who reported relatively fewer PSU symptoms to endorse a relatively higher level of pandemic-related self-efficacy, perceived pandemic-related government efficacy, and reward for application; in contrast, those who reported relatively more PSU symptoms are expected to endorse a relatively higher level of social cynicism. Due to the PSU data acquired from a probability Chinese adult community sample, we hoped to shed some light on the development of PSU during the global crisis. The pandemic-related beliefs and generalized social beliefs under study help to address the missing link of how these genres of beliefs are related to the severity of PSU during the pandemic and may also identify some promising directions for studying potential correlates of other addictive behaviors that may develop during the pandemic. These findings may provide practical insights for PSU screening in the Chinese community and point out directions in designing cognitive interventions and preventions for PSU during the pandemic.

## 2. Methods

### 2.1. Procedures and Participants

This study adopted a telephone survey with a two-stage cluster random sampling method to collect data from adults (age 18 or above) among Chinese community dwellers of both genders, in Macao, China, in April 2020. We randomly selected households as clusters in the first stage, and then performed the second-stage random selection of one participant within each household based on the last birthday rule (i.e., the one who most recently had their birthday was chosen [46]). Trained research assistants briefed the selected participants on the nature of the present study and their rights of participation in the study. Only respondents who gave oral consent to participate voluntarily and without monetary incentives received the formal survey interview. This study obtained ethical approval from the research ethics committee of the corresponding author’s affiliated department.

In total, 616 participants (39.1% men; 95% CI [35.2%, 43.0%]), ranging in age from 18 to 87 years (*M* = 41.7, *SD* = 16.3), completed the telephone survey. The overall cooperation rate was 89.9%, which was calculated according to the formula proposed by the American Association for Public Opinion Research (i.e., using all cases interviewed divided by all eligible respondents ever contacted; 2016). Twenty-five respondents who reported not having a smartphone were excluded and the data of the remaining 591 participants (39.4% men; *M_age_* = 40.5 years old, *SD_age_* = 15.3) were included for the formal analysis. Regarding levels of education, half of the participants received education at the tertiary level, followed by senior high (26.1%), junior high (12.0%), and primary school or below (6.8%) levels. No participants had a prior infection of COVID-19.

### 2.2. Measures

#### 2.2.1. PSU

Respondents’ PSU over the past month was measured by the Chinese version of Kwon et al.’s [47] Smartphone Addiction Scale-Short Version (SAS-SV) [48]. The 10-item scale (e.g., “Missing planned work due to smartphone use”) was rated and scored on a 6-point Likert scale (1 = *Strongly disagree* to 6 = *Strongly agree*), with the total score ranging from 10 to 60. A higher scale sum score indicated more PSU symptoms and hence a higher risk for PSU. The cutoff score was 31 for male and 33 for female participants [47]. The internal consistency of this scale, assessed by Cronbach’s alpha, was 0.88 in this study.

#### 2.2.2. Social Axioms

Two subscales of the Social Axioms Survey [38], social cynicism and reward for application, were used in this study. Participants responded to two 8-item subscales on a 5-point Likert scale, in which 1 = *strongly disbelieve* and 5 = *strongly believe*. A sample item of social cynicism is “kind-hearted people usually suffer losses,” whereas a sample item of reward for application is “building the way step by step leads to success.” A higher score represented a higher level of the corresponding social axiom construct. Social cynicism (α = 0.80) and reward for application (α = 0.88) both showed a satisfactory internal consistency in this sample.

#### 2.2.3. Pandemic-Related Self-Efficacy

Pandemic-related self-efficacy was assessed by one item related to participants’ perceived self-efficacy toward adopting COVID-19 precautionary measures: “I believe I have the ability to comply with COVID-19 precautionary measures recommended by the government”; the item was measured on a 5-point Likert scale, which ranged from 1 = *strongly disagree* to 5 = *strongly agree*. A similar 1-item self-efficacy assessment had been previously adopted in epidemic and pandemic scenarios [24,49]. A higher score referred to a higher level of self-efficacy.

#### 2.2.4. Perceived Pandemic-Related Government Efficacy

Perceived pandemic-related government efficacy entailed one’s perception of the local government’s capability of pandemic control and was measured by one item: “How confident are you that the government can successfully combat the COVID-19 pandemic?” The item was rated on a 5-point Likert scale in which 1 = *very unconfident* and 5 = *very confident*. A similar item was used in previous studies, such as measuring the Singaporean response to the outbreak of severe acute respiratory syndrome [50]. A higher score represented a higher level of belief in the government’s precautionary measures.

#### 2.2.5. Demographic Information

Demographic information on gender (0 = *female*, 1 = *male*), age, and educational level (1 = *no formal education*, 2 = *primary school*, 3 = *junior high school*, 4 = *senior high school*, 5 = *junior college diploma*, and 6 = *bachelor or above*) were collected in the survey. Additional COVID-19 related data were gathered regarding whether the respondents had been infected with COVID-19.

### 2.3. Data Analysis

All statistical analyses were conducted with SPSS 24. The descriptive statistics and bivariate correlations of all key variables were examined first. Five hierarchical multiple regressions, Models A to E, were conducted to further investigate the associations among pandemic-specific beliefs, generalized beliefs, and PSU. In all five models, gender, age, and educational level were controlled for as covariates in the first step. Each of the two pandemic-related beliefs was entered in Model A and B, respectively. Similarly, each of the generalized social beliefs was independently entered in Model C and D in the second step. As for Model E, we entered all four types of beliefs together in the second step. By comparing the results of the five models, we may infer the relative strength of the four different beliefs in relation to PSU. Additionally, we also checked the linear regression assumptions of residual normality, homoscedasticity, linearity, error impendence, and absence of unexpected outliers with plotting standardized residuals against standardized predicted values (i.e., looking for a random array of dots; [51]), histogram and normal probability plot of standardized residuals (i.e., normal distribution in histogram and dots lining along the diagonal line in probability plot; [51]), and Cook’s distance test (i.e., smaller than 1; [52]).

## 3. Results

### 3.1. Descriptive and Correlation Analyses

The descriptive statistics of the core variables and their bivariate correlation coefficients are shown in Table 1. The prevalence of PSU was 43.3% in the overall sample; 41.9% in women, and 45.5% in men. Although we identified more male than female respondents who might be at a risk for PSU, this difference was not statistically significant, χ^2^(1) = 0.74, *p* = 0.39. Similarly, the mean score difference between male (*M* = 30.15, *SD* = 9.74) and female respondents (*M* = 29.62, *SD* = 10.35) was statistically nonsignificant, *t*(589) = 0.62, *p* = 0.53. All four hypothesized correlates with PSU were significant in expected directions. Specifically, pandemic-related self-efficacy and pandemic-related government efficacy had a negative correlation with PSU (*r* = −0.13 and –0.10, *p* < 0.05). Reward for application was negatively correlated with PSU (*r* = −0.10, *p* < 0.05), whereas social cynicism was positively associated with PSU (*r* = 0.25, *p* < 0.001). Moreover, reward for application had positive associations with pandemic-related self-efficacy and perceived pandemic-related government efficacy at a significant level (*r* = 0.20 and 0.27, respectively, *p* < 0.001). As for demographics, gender had no significant correlation with any of the core variables. Age was inversely correlated with PSU (*r* = −0.22, *p* < 0.001), but positively correlated with pandemic-related government efficacy as well as reward for application (*r* = 0.25 and 0.28, respectively, *p* < 0.001). Educational level was negatively correlated with pandemic-related government efficacy, social cynicism, and reward for application at a significant level (*r* = −0.10 to −0.18, *p* < 0.05).

### 3.2. Hierarchical Multiple Regressions on PSU

All the assumptions of multiple linear regressions were held for all five models. As shown in Table 2, the first four hierarchical multiple regressions, Models A to D, each independently tested the relationship between one belief and PSU after controlling for gender, age, and educational level. Regarding the two pandemic-related beliefs, government efficacy accounted for a significant addition (0.8%) of the variance in PSU (Δ*F* = 4.82, *p* = 0.03) beyond the variance explained by demographic variables; in contrast, self-efficacy only explained a marginally significant increment (0.6%) of the variance in PSU (Δ*F* = 3.34, *p* = 0.07). As for the two generalized social beliefs, social cynicism explained an additional 6.8% of the variance in PSU, which was at a significant level (Δ*F* = 42.62, *p* < 0.001), whereas reward for application did not bring a significant increase in the explained variance (Δ*F* = 1.18, *p* = 0.28). When entering all four beliefs at the same time in Model E, this second step resulted in a significant increment of 7.3% of the variance explained in PSU (Δ*F* = 11.37, *p* < 0.001). Social cynicism was identified as the only significant predictor in this step (*β* = 0.25, *p* < 0.001), indicating that social cynicism is the most prominent correlate of PSU among the four beliefs.

## 4. Discussion

In this study, we observed a high prevalence of probable PSU, as high as 43.3% among Chinese adults during the pandemic period, which is higher than previous estimates based on another Chinese adult sample (i.e., 38.5% [48]). The difference was plausibly attributed to greater reliance on Internet devices for various purposes during the pandemic, which made people more vulnerable to PSU. As shown in recent research, the increased number of COVID-19 infected cases has consistently led to more online searches regarding protective behaviors, health knowledge, and panic buying across four countries [53]. Given that the smartphone is the most widely used device by Chinese netizens to access the Internet (i.e., 99.3%; compared to 42.7% desktop computers and 35.1% laptops [54]), we may expect that a large proportion of the increase in Internet searches concerning COVID-19 would be performed in smartphones among Chinese netizens. Moreover, stay-at-home mandates/recommendations and quarantines allow individuals to spend more time on their smartphones [55].

Consistent with previous studies [48,56], younger respondents reported significantly more PSU symptoms and a higher risk for probable PSU in our sample than their older counterparts. Unlike some extant findings that have pointed out that females may be more prone to PSU [44,48], we did not find a statistically significant gender difference regarding the risk for PSU. Admittedly, various possible explanations may exist for this unfound gender difference in our study, and we speculate that the pandemic may have changed smartphone use patterns among men. In past research, women were found to be more motivated to keep up social connections through smartphone use than men during pre-pandemic times [57]; however, when the pandemic discouraged face-to-face interactions, men may have been more motivated to rely on the smartphone for social interactions. The effect of educational attainment on PSU was not salient in the present study, and hence the pandemic may have put people from all educational levels under risk for PSU.

Supporting H1 and H2, pandemic-related self-efficacy and government efficacy both displayed a significant association with PSU in a negative direction, indicating that the two beliefs may play a potentially protective role in lowering one’s propensity for PSU. After controlling for demographic effects (i.e., gender, age, and educational level), the pandemic-related government efficacy had a statistically significant and negative association with PSU. These findings may imply that pandemic-related government efficacy serves as a protective factor against the development of PSU among Chinese adults regardless of their gender, age, and educational level. The findings are also consistent with previous empirical evidence that pandemic-related self-efficacy bears a potential effect on promoting adherence to precautionary measures and lowering psychological distress during the pandemic [58,59]. However, pandemic-related self-efficacy did not significantly add to the explained variance in PSU beyond the demographic effects. We may cautiously infer from this result that the pandemic-related self-efficacy effect on PSU is relatively more variant across different genders, ages, and educational levels. Between the two forms of efficacy, government efficacy appears to generate a protective effect against PSU in a relatively more reliable way. It is plausible that a higher level of pandemic-related government efficacy is more consistently associated with a lower level of mental health burden and hence would be associated with a lower need to turn to maladaptive coping methods, such as PSU.

As for the two generalized social beliefs, according to the bivariate correlation results, these two beliefs were significantly correlated with PSU in expected directions, consistent with H3 and H4. People who held high levels of social cynicism tended to be exposed to a higher risk for PSU, and those who reported a stronger belief in reward for application appeared to be under a lower PSU risk. When taking the demographic effect into account, social cynicism contributed to explaining additional variance in PSU, while reward for application did not. It seems that social cynicism’s exacerbating effect on PSU is more consistent in the face of variations in gender, age, and educational level. It is plausible that people who hold more negative views towards government and authorities would experience a higher level of psychological burden in the midst of the pandemic, which would contribute to a heightened risk for PSU.

When putting all the four beliefs together, social cynicism exerted the most substantial effect on PSU beyond the demographic effects. Individuals with higher levels of social cynicism tend to attribute mistakes or problems to others and environments, and so they are most likely to engage in wishful thinking and maladaptive coping [43]. During the pandemic, individuals who hold stronger beliefs in social cynicism may be more prone to excessive use of smartphone to regulate negative emotions and/or pass time, and excessive smartphone use is one of the common maladaptive coping strategies under stressful conditions [44]. Given the negative influence of social cynicism on psychological wellbeing (e.g., [39]), our findings enriched the empirical evidence that individuals who are more inclined to social cynicism are more vulnerable to PSU during the pandemic.

Although the present study made the first attempt to investigate the potential relationship between different genres of beliefs and PSU during the pandemic, it is limited in several respects. First, due to its cross-sectional nature, we interpreted the results on the correlational level. Subsequent studies are encouraged to use longitudinal or experimental designs based on our current findings to track changes over time or make causal inferences between beliefs and PSU. Second, as we only tested how two genres of beliefs were related to PSU, our coverage of COVID-19 related cognitions is limited. We encourage interested parties to extend the exploration to relationships between other types of cognitions and PSU during the pandemic. Third, our study did not directly control for the effects of affective or motivational constructs, such as mental distress, which can be a potential mediator between cognitions and PSU. Future studies may include such constructs to deepen our understanding of the psychological mechanisms underlying the relationships between beliefs and PSU. Lastly, other demographic variables, such as socioeconomic status, can be controlled for in future studies to minimize any potential confounding effects.

## 5. Conclusions

To understand the implications of the increased smartphone use in the general public during the COVID-19 pandemic, the present study estimated the prevalence of PSU within the Chinese adult community using a probability sampling method. Findings showed an elevated risk of PSU among the Chinese adults. As PSU risk may be triggered and fueled by the pandemic’s development, we identified the effect of two pandemic-related beliefs and two generalized social beliefs on PSU during the pandemic. Based on the current findings, pandemic-specific PSU interventions that take into account the effect of these four beliefs on PSU could be designed. Pandemic-related government efficacy and social cynicism appeared to be more invariantly associated with PSU despite potential variations in gender, educational level, and age, whereas social cynicism displayed the most potent effect on PSU among the four beliefs. These findings enriched our understanding of PSU development and may be useful in informing cognition-based intervention strategies for reducing PSU. Moreover, our findings point to potentially promising research directions for behavioral addictions during the pandemic.

## Figures and Tables

**Table 1:** Descriptive statistics and bivariate correlation among the core constructs (*N* = 591).

	M	SD	1	2	3	4	5	6	7
1.PSU	29.83	10.11	1						
2. Pandemic-related self-efficacy	4.25	0.73	−0.10 *	1					
3. Perceived pandemic-related government efficacy	4.28	0.61	−0.13 **	0.27 ***	1				
4. Social cynicism	2.85	0.68	0.25 ***	−0.04	−0.12 **	1			
5. Reward for application	3.85	0.64	−0.10 *	0.20 ***	0.27 ***	0.002	1		
6. Educational Level	4.70	1.31	0.09 ^†^	0.02	−0.12 **	−0.10 *	−0.18 ***	1	
7. Age	40.46	15.32	−0.22 ***	0.05	0.25 ***	0.03	0.28 ***	−0.52 ***	1
8. Gender	0.39	0.49	0.03	−0.05	−0.04	0.08	−0.05	−0.01	−0.08 ^†^

Note: PSU: problematic smartphone use. * *p* < 0.05; ** *p* < 0.01; *** *p* < 0.001; ^†^ *p* < 0.05 but *r* value does not reach a small effect size (lower than 0.10).

**Table 2:** Five alternative hierarchical multiple regressions on PSU.

Variable	PSU		
	*b* (SE)	*β* [95% CI]	*p*
**Step 1**			
Gender	0.12(0.86)	0.01[−1.56, 1.80]	0.89
Age	−0.15(0.03)	−0.22[−0.21, −0.08]	<0.001
Educational level	−0.14(0.37)	−0.02[−0.87, 0.60]	0.72
*F*/*R*^2^	8.60/0.045		<0.001
**Step 2**			
**Model A**			
Pandemic-related self-efficacy	−1.06 (0.58)	−0.08[−2.20, 0.08]	0.07
Δ*F*/Δ*R*^2^	3.34/0.006		0.07
**Model B**			
Perceived pandemic-related government efficacy	−1.55(0.71)	−0.09[−2.93, −0.16]	0.03
Δ*F*/Δ*R*^2^	4.82/0.008		0.03
**Model C**			
Social cynicism	3.98(0.61)	0.26[2.78, 5.18]	<0.001
Δ*F*/Δ*R*^2^	42.62/0.068		<0.001
**Model D**			
Reward for application	−0.76(0.70)	−0.05[−2.14, 0.62]	0.28
Δ*F*/Δ*R*^2^	1.18/0.002		0.28
**Model E**			
Pandemic-related self-efficacy	−0.81(0.58)	−0.06[−1.95, 0.34]	0.17
Perceived pandemic-related government efficacy	−0.63(0.73)	−0.04 [−2.05, 0.80]	0.39
Social cynicism	3.78(0.61)	0.25[2.58, 4.99]	<0.001
Reward for application	−0.39(0.70)	−0.03[−1.76, 0.98]	0.57
Δ*F*/Δ*R*^2^	11.37/0.073		<0.001

Note: PSU: problematic smartphone use.

## Data Availability

The data presented in this study are available on request from the corresponding author.

## References

[1] World Health Organization Listings of WHO’s Response to COVID-19. https://www.who.int/news/item/29-06-2020-covidtimeline.

[2] World Health Organization Coronavirus Disease (COVID-19) Advice for the Public. https://www.who.int/emergencies/diseases/novel-coronavirus-2019/advice-for-public.

[3] Collado-Borrell R., Escudero-Vilaplana V., Villanueva-Bueno C., Herranz-Alonso A., Sanjurjo-Saez M. (2020). Features and Functionalities of Smartphone Apps Related to COVID-19: Systematic Search in App Stores and Content Analysis. J. Med. Internet Res..

[4] Iyengar K., Upadhyaya G.K., Vaishya R., Jain V. (2020). COVID-19 and applications of smartphone technology in the current pandemic. Diabetes Metab. Syndr. Clin. Res. Rev..

[5] Ohme J., Abeele M.M.P.V., Van Gaeveren K., Durnez W., De Marez L. (2020). Staying Informed and Bridging “Social Distance”: Smartphone News Use and Mobile Messaging Behaviors of Flemish Adults during the First Weeks of the COVID-19 Pandemic. Socius Sociol. Res. Dyn. World.

[6] Masaeli N., Farhadi H. (2021). Prevalence of Internet-based addictive behaviors during COVID-19 pandemic: A systematic review. J. Addict. Dis..

[7] Elhai J.D., Yang H., McKay D., Asmundson G.J. (2020). COVID-19 anxiety symptoms associated with problematic smartphone use severity in Chinese adults. J. Affect. Disord..

[8] Billieux J. (2012). Problematic Use of the Mobile Phone: A Literature Review and a Pathways Model. Curr. Psychiatry Rev..

[9] Kuss D.J., Harkin L., Kanjo E., Billieux J. (2018). Problematic Smartphone Use: Investigating Contemporary Experiences Using a Convergent Design. Int. J. Environ. Res. Public Health.

[10] Gao J., Zheng P., Jia Y., Chen H., Mao Y., Chen S., Wang Y., Fu H., Dai J. (2020). Mental health problems and social media exposure during COVID-19 outbreak. PLoS ONE.

[11] Xiong J., Lipsitz O., Nasri F., Lui L.M.W., Gill H., Phan L., Chen-Li D., Iacobucci M., Ho R., Majeed A. (2020). Impact of COVID-19 pandemic on mental health in the general population: A systematic review. J. Affect. Disord..

[12] Coyne S.M., Stockdale L., Summers K. (2019). Problematic cell phone use, depression, anxiety, and self-regulation: Evidence from a three year longitudinal study from adolescence to emerging adulthood. Comput. Hum. Behav..

[13] Mehroof M., Griffiths M.D. (2010). Online Gaming Addiction: The Role of Sensation Seeking, Self-Control, Neuroticism, Aggression, State Anxiety, and Trait Anxiety. Cyberpsychol. Behav. Soc. Netw..

[14] Savolainen I., Kaakinen M., Sirola A., Oksanen A. (2018). Addictive behaviors and psychological distress among adolescents and emerging adults: A mediating role of peer group identification. Addict. Behav. Rep..

[15] Wu A.M.S., Lau J.T.F., Cheng K.-M., Law R.W., Tse W.S., Lau M.M.C. (2018). Direct and Interaction Effects of Co-Existing Familial Risk Factors and Protective Factors Associated With Internet Addiction Among Chinese Students in Hong Kong. J. Early Adolesc..

[16] Eisenberg D., Speer N., Hunt J.B. (2012). Attitudes and Beliefs About Treatment Among College Students with Untreated Mental Health Problems. Psychiatr. Serv..

[17] Bandura A., Vasta R. (1989). Social cognitive theory. Annals of Child Development.

[18] Schwarzer R., Luszczynska A. (2008). How to Overcome Health-Compromising Behaviors. Eur. Psychol..

[19] Luszczynska A., Benight C.C., Cieslak R. (2009). Self-Efficacy and Health-Related Outcomes of Collective Trauma. Eur. Psychol..

[20] Mystakidou K., Tsilika E., Parpa E., Gogou P., Theodorakis P., Vlahos L. (2010). Self-efficacy beliefs and levels of anxiety in advanced cancer patients. Eur. J. Cancer Care.

[21] Wu A.M.S., Lai M.H.C., Lau J.T.F., Walden D.L. (2020). Incidence of Probable Depression and Its Predictors Among Chinese Secondary School Students. Int. J. Ment. Health Addict..

[22] Lau J.T.F., Ng C.S.M., Wu A.M.S., Ma Y.L., Lau M.M.C. (2018). Low coverage of influenza vaccination among Chinese children aged 12–23 months: Prevalence and associated factors. PLoS ONE.

[23] Lau J.T.F., Wu A.M.S., Gross D.L., Cheng K.-M., Lau M.M.C. (2017). Is Internet addiction transitory or persistent? Incidence and prospective predictors of remission of Internet addiction among Chinese secondary school students. Addict. Behav..

[24] Yıldırım M., Güler A. (2020). COVID-19 severity, self-efficacy, knowledge, preventive behaviors, and mental health in Turkey. Death Stud..

[25] Tang C.S.-K., Wu A.M.S. (2010). Direct and Indirect Influences of Fate Control Belief, Gambling Expectancy Bias, and Self-Efficacy on Problem Gambling and Negative Mood Among Chinese College Students: A Multiple Mediation Analysis. J. Gambl. Stud..

[26] Tang C.S.-K., Wu A.M.S. (2011). Gambling-Related Cognitive Biases and Pathological Gambling Among Youths, Young Adults, and Mature Adults in Chinese Societies. J. Gambl. Stud..

[27] Wu A.M.S., Li J., Lau J.T.F., Mo P.K.H., Lau M.M.C. (2016). Potential impact of internet addiction and protective psychosocial factors onto depression among Hong Kong Chinese adolescents—Direct, mediation and moderation effects. Compr. Psychiatry.

[28] Bandura A. (1977). Self-efficacy: Toward a unifying theory of behavioral change. Psychol. Rev..

[29] Wang C., Pan R., Wan X., Tan Y., Xu L., McIntyre R.S., Choo F.N., Tran B., Ho R., Sharma V.K. (2020). A longitudinal study on the mental health of general population during the COVID-19 epidemic in China. Brain Behav. Immun..

[30] Emirtekin E., Balta S., Sural I., Kircaburun K., Griffiths M.D., Billieux J. (2019). The role of childhood emotional maltreatment and body image dissatisfaction in problematic smartphone use among adolescents. Psychiatry Res..

[31] Chong Y.Y., Chien W.T., Cheng H.Y., Chow K.M., Kassianos A.P., Karekla M., Gloster A. (2020). The Role of Illness Perceptions, Coping, and Self-Efficacy on Adherence to Precautionary Measures for COVID-19. Int. J. Environ. Res. Public Health.

[32] Roma P., Monaro M., Muzi L., Colasanti M., Ricci E., Biondi S., Napoli C., Ferracuti S., Mazza C. (2020). How to Improve Compliance with Protective Health Measures during the COVID-19 Outbreak: Testing a Moderated Mediation Model and Machine Learning Algorithms. Int. J. Environ. Res. Public Health.

[33] Macao SAR Centre for Disease Control and Prevention Special Webpage against Epidemics: Preventive Measures. https://www.ssm.gov.mo/apps1/PreventCOVID-19/en.aspx#clg17048.

[34] Saechang O., Yu J., Li Y. (2021). Public Trust and Policy Compliance during the COVID-19 Pandemic: The Role of Professional Trust. Health.

[35] Van Der Weerd W., Timmermans D.R., Beaujean D.J., Oudhoff J., Van Steenbergen J.E. (2011). Monitoring the level of government trust, risk perception and intention of the general public to adopt protective measures during the influenza A (H1N1) pandemic in the Netherlands. BMC Public Health.

[36] Bäuerle A., Teufel M., Musche V., Weismüller B., Kohler H., Hetkamp M., Dörrie N., Schweda A., Skoda E.-M. (2020). Increased generalized anxiety, depression and distress during the COVID-19 pandemic: A cross-sectional study in Germany. J. Public Health.

[37] Leung K., Bond M.H. (2004). Social Axioms: A Model for Social Beliefs in Multicultural Perspective. Adv. Exp. Soc. Psychol..

[38] Leung K., Lam B.C.P., Bond M.H., Conway I.L.G., Gornick L.J., Amponsah B., Boehnke K., Dragolov G., Burgess S.M., Golestaneh M. (2011). Developing and Evaluating the Social Axioms Survey in Eleven Countries. J. Cross-Cult. Psychol..

[39] Li Y., Tong K.K., Tao V.Y.K., Zhang M.X., Wu A.M.S. (2020). Testing the Associations among Social Axioms, School Belonging, and Flourishing in University Students: A Two-Year Longitudinal Study. Appl. Psychol. Health Well-Being.

[40] Chen S.X., Lam B.C.P., Wu W.C.H., Ng J.C.K., Buchtel E.E., Guan Y., Deng H. (2016). Do people’s world views matter? The why and how. J. Pers. Soc. Psychol..

[41] Neto F. (2006). Dimensions and Correlates of Social Axioms Among a Portuguese Sample. Individ. Differ. Res..

[42] Wu W.C.H., Chen S.X., Wong S.S.-K. (2019). Predicting Gambling Propensity and Behavior: The Role of Social Axioms and Distortive Beliefs. J. Gambl. Stud..

[43] Bond M.H., Leung K., Au A., Tong K.-K., De Carrasquel S.R., Murakami F., Yamaguchi S., Bierbrauer G., Singelis T.M., Broer M. (2004). Culture-Level Dimensions of Social Axioms and Their Correlates across 41 Cultures. J. Cross-Cult. Psychol..

[44] Long J., Liu T.-Q., Liao Y.-H., Qi C., He H.-Y., Chen S.-B., Billieux J. (2016). Prevalence and correlates of problematic smartphone use in a large random sample of Chinese undergraduates. BMC Psychiatry.

[45] Lam B.C.P., Bond M.H., Chen S.X., Wu W.C.H. (2010). Worldviews and Individual Vulnerability to Suicide: The Role of Social Axioms. Eur. J. Pers..

[46] Gaziano C., Lavrakas P. (2013). Last-Birthday Selection. Encyclopedia of Survey Research Methods.

[47] Kwon M., Kim D.-J., Cho H., Yang S. (2013). The Smartphone Addiction Scale: Development and Validation of a Short Version for Adolescents. PLoS ONE.

[48] Luk T.T., Wang M.P., Shen C., Wan A., Chau P.H., Oliffe J., Viswanath K., Chan S.S.-C., Lam T.H. (2018). Short version of the Smartphone Addiction Scale in Chinese adults: Psychometric properties, sociodemographic, and health behavioral correlates. J. Behav. Addict..

[49] De Zwart O., Veldhuijzen I.K., Elam G., Aro A.R., Abraham T., Bishop G.D., Voeten H.A.C.M., Richardus J.H., Brug J. (2009). Perceived Threat, Risk Perception, and Efficacy Beliefs Related to SARS and Other (Emerging) Infectious Diseases: Results of an International Survey. Int. J. Behav. Med..

[50] Deurenberg-Yap M., Foo L.L., Low Y.Y., Chan S.P., Vijaya K., Lee M. (2005). The Singaporean response to the SARS outbreak: Knowledge sufficiency versus public trust. Health Promot. Int..

[51] Field A. (2018). Discovering Statistics Using IBM SPSS Statistics.

[52] Cook R.D., Weisberg S. (1982). Residuals and Influence in Regression.

[53] Du H., Yang J., King R.B., Yang L., Chi P. (2020). COVID-19 Increases Online Searches for Emotional and Health-Related Terms. Appl. Psychol. Health Well-Being.

[54] China Internet Network Information Center The 45th China Statistical Report on Internet Development. https://cnnic.com.cn/IDR/ReportDownloads/.

[55] Sañudo B., Fennell C., Sánchez-Oliver A. (2020). Objectively-Assessed Physical Activity, Sedentary Behavior, Smartphone Use, and Sleep Patterns Pre- and during-COVID-19 Quarantine in Young Adults from Spain. Sustainability.

[56] Nahas M., Hlais S., Saberian C., Antoun J. (2018). Problematic smartphone use among Lebanese adults aged 18–65 years using MPPUS-10. Comput. Hum. Behav..

[57] Desouky D.E.-S., Abu-Zaid H. (2020). Mobile phone use pattern and addiction in relation to depression and anxiety. East. Mediterr. Health J..

[58] Bendau A., Plag J., Kunas S., Wyka S., Ströhle A., Petzold M.B. (2021). Longitudinal changes in anxiety and psychological distress, and associated risk and protective factors during the first three months of the COVID-19 pandemic in Germany. Brain Behav..

[59] Bressington D.T., Cheung T.C.C., Lam S.C., Suen L.K.P., Fong T.K.H., Ho H.S.W., Xiang Y.-T. (2020). Association Between Depression, Health Beliefs, and Face Mask Use During the COVID-19 Pandemic. Front. Psychiatry.

